# Calox, the New Dentifrice

**Published:** 1904-06

**Authors:** 


### Calox, the New Dentifrice.

MORE knowledge relating to the care of the teeth and less indifference regarding its importance would make the human race healthier and longevity would be increased. The occupation of the dentist would then be limited to a somewhat narrower field than he occupies at present, but it would be none the less dignified. To overcome tooth decay, the dentist must be something more than a mere cleaner of teeth, though the importance of this latter function should not be decried. To clean the teeth properly is an art that is not acquired by every dentist.

Since the discovery of the real causes of tooth decay only ignorant or dishonest dentists impeach the integrity or skill of the medical profession, by telling their patrons that strong and irritant medicines have caused the difficulty. Every educated dentist knows that tooth decay is due to the local action of lactic acid, produced through the agency of specific kinds of bacteria, which find a suitable field of growth in the human mouth. It is also generally conceded by authorities in dental pathology, that erosions of the teeth causing extensive loss of structure are due also to acid action, either by acid fermentation in the mouth or by acidity of the secretions of the mucous glands of the mouth.

When, therefore, a dentist is heard charging the administration of "strong medicines" with causing the early decay of teeth, he should be placed on the list of ignoramuses, or in the category of knaves. He betrays either ignorance or wilful depravity.

It was not our purpose, however, to discuss this phase of the subject when we began this writing, but we have been led into it by mere correlation of thought. To return, then, to our original purpose, we invite attention to the fact that a new dentifrice has been presented to the public through both medical and dental professions,. which promises to accomplish what others have failed to do—namely, sterilise the mouth, arrest decay, and at the same time prove both agreeable and harmless. The name of this new dentifrice is *calox,*—a calcium dioxide added in appropriate proportions to English precipitated chalk as a basis, and which is then rendered very slightly saponacious and delicately flavored. It is to calcium dioxide that this new dentifrice owes its cleansing and germicidal properties. Of the value of the latter chemists say that calcium dioxide, like hydrogen dioxide, has the property of giving up oxygen in the nascent state when brought into contact with water or dead organic matter, at the same time forming milk of lime; it is, therefore, a powerful oxidising agent, germicide and antacid. It is quickly and completely decomposed by all acids, thus forming a salt of calcium and setting free hydrogen dioxide.

Mr. E. H. Gane, a chemist of experience, read a paper recently before the New York Odontological Society, in which he discussed the dental applications of some of the new oxygen compounds. An abstract of this paper is presented elsewhere, and will prove of interest to every person who desires to preserve sound and wholesome teeth.

The sum of the whole matter would seem definitely to indicate that when this new dentifrice, calox, is brought into contact with the fluids of the mouth, oxygen is at once liberated, which, while without effect on the soft tissues of the mouth, is an effective destroyer of decomposing organic matter and bacterial life. In contact with the local acid areas found in decay of the teeth, erosion, or acid accumulations of fermenting food particles between or upon the teeth, these local acids decompose the calcium dioxide of the dentifrice, which neutralises their acidity by entering into combination with the calcium of the dioxide and setting free hydrogen dioxide, to exercise its well-known germicidal and detergent effects upon the bacteria and dead organic matter present. Because of this chemical reaction of calcium dioxide with acids, calox posseses a selective property for those situations on and about the teeth where local acid areas exist, and its oxidising, neutralising and germicidal powers are therefore exerted to the greatest extent where there is most necessity for such action.

This interesting subject might be pursued to a greater length, but we have said enough to point out its importance and to indicate a remedy,—a joint product of chemical and dental science,—for one of the greatest evils of our modern civilisation.

